# Patterns of Human Papillomavirus Types in Multiple Infections: An Analysis in Women and Men of the High Throughput Human Papillomavirus Monitoring Study

**DOI:** 10.1371/journal.pone.0071617

**Published:** 2013-08-19

**Authors:** Salvatore Vaccarella, Anna Söderlund-Strand, Silvia Franceschi, Martyn Plummer, Joakim Dillner

**Affiliations:** 1 International Agency for Research on Cancer, Lyon, France; 2 Department of Clinical Microbiology, Skåne University Hospital, Malmö, Sweden; 3 Departments of Laboratory Medicine, Medical Epidemiology and Biostatistics, Karolinska Institutet and Karolinska Hospital, Stockholm, Sweden; The Catalan Institute of Oncology (ICO), Spain

## Abstract

**Background:**

To evaluate the pattern of co-infection of human papillomavirus (HPV) types in both sexes in Sweden.

**Methods:**

Cell samples from genital swabs, first-void urine, and genital swabs immersed in first-void urine were collected in the present cross-sectional High Throughput HPV Monitoring study. Overall, 31,717 samples from women and 9,949 from men (mean age 25) were tested for 16 HPV types using mass spectrometry. Multilevel logistic regression was used to estimate the expected number of multiple infections with specific HPV types, adjusted for age, type of sample, and accounting for correlations between HPV types due to unobserved risk factors using sample-level random effects. Bonferroni correction was used to allow for multiple comparisons (120).

**Results:**

Observed-to-expected ratio for any multiple infections was slightly above unity in both sexes, but, for most 2-type combinations, there was no evidence of significant departure from expected numbers. HPV6/18 was found more often and HPV51/68 and 6/68 less often than expected. However, HPV68 tended to be generally underrepresented in co-infections, suggesting a sub-optimal performance of our testing method for this HPV type.

**Conclusions:**

We found no evidence for positive or negative clustering between HPV types included in the current prophylactic vaccines and other untargeted oncogenic types, in either sex.

## Introduction

Persistent infection with human papillomavirus (HPV), a common sexually transmitted infection, is considered a necessary cause of cervical cancer and is also associated with other anogenital cancers in men and women [1]. Genital infection with multiple HPV types is commonly found in women [2]–[12] and men [13]–[17].

Current prophylactic HPV vaccines target the two most oncogenic types, HPV16 and 18, and one of the two vaccines also targets two non-oncogenic types, HPV6 and 11. A possibility exists that the decrease of certain HPV types through vaccination could lead to a shift in the distribution of HPV genotypes, with an increase (replacement) or decrease (on account of cross-protection [18]) in the prevalence of non-targeted HPV types in the population. This issue can only be fully answered by prospective studies of vaccinated populations. However, some information on whether such changes are likely or not can be gained by studying infections with multiple HPV types, and using a statistical model to determine whether the prevalence of multiple infections can be explained entirely by their shared sexual transmission and common risk factors.

Previous analyses on the frequency of individual HPV types in co-infections with multiple types suggest that they do not tend to cluster together selectively in either sex [2], [3], [7], [8], [11], [16], [19]. Some studies reported that co-infections with certain HPV types occurred more often than what was expected, but this was explained by diagnostic artefacts due to limitations of the HPV detection method used [2], [16]. Overall, multiple co-infections show no specific pattern by HPV type. However, a full evaluation of the analysis of 2-type associations between all the different HPV types requires a large number of infections and should preferably include men and women. In the present analysis, therefore, we evaluated the patterns of HPV types in co-infections in women and men in a large study, the High Throughput HPV Monitoring (HT-HPV) study.

## Methods

### Study Population

The HT-HPV study was a survey of HPV prevalence among young sexually active women and men in Southern Sweden. The aim of the HT-HPV study was to establish baseline data to monitor the input of HPV vaccination. The study was based on participants in the *Chlamydia trachomatis* testing programme, which mainly recruited participants from youth clinics for sexual health counselling; gynecology clinics/maternal care units (women only); venereology/urology clinics; and primary care units. Samples collected for *Chlamydia trachomatis* testing were also analyzed for HPV DNA. All samples were anonymized and age and sex are the only information available for study participants. The Regional Ethical Review Board for Southern Sweden approved the study and decided that informed consent for HPV testing was not required, since samples were anonymized. We cannot, therefore, identify which samples are repeat tests on the same individuals and the total number of participants is not known exactly. Although the study size is reported in terms of samples, not individuals, it is estimated that the number of unique individuals is 78% of the number of samples [20].

Overall, 44,146 urine and swab samples were collected (33,137 from women and 11,009 from men) between March and October 2008 using the multi-Collect Specimen Collection Kit and following the manufacturer’s instructions (Abbott Molecular, Illinois, USA). The present analysis is restricted to individuals aged 12–45 years. In addition, as *Chlamydia trachomatis* testing is performed on samples from various sites of the body, the present analysis is restricted to urogenital samples, with samples taken from the rectum, eye, pharynx and other sites being excluded. These restrictions leave a total of 31,717 samples from women, of which 13,347 were genital swabs immersed in first-void urine, 10,624 first-void urine and 7,746 genital swabs (7,024 from cervix, 637 from cervix or urethra, 45 from vagina and 40 from urethra). From men, 9,949 samples were available, of which were 9,360 first-void urine, 535 urethral swabs and 54 genital swabs immersed in first-void urine.

### HPV Testing and Genotyping

DNA was extracted for *Chlamydia trachomatis* testing using the Abbott m2000sp system according to the manufacturer’s instructions (Abbott Molecular, Illinois, USA), the residual DNA was tested for HPV6, 11, 16, 18, 31, 33, 35, 39, 45, 51, 52, 56, 58, 59, 66, and 68, using polymerase chain reaction (PCR) followed by matrix-assisted laser desorption ionization-time of flight mass spectrometry (MS), as previously described [20]. Briefly, the MS method involves a consensus PCR reaction using the MGP primer system [21] followed by a mass extension (ME) reaction with a single ME primer of distinct mass that is specific for each HPV genotype. After completion of the ME reaction, unextended primers demonstrate the absence and extended primers the presence and identity of each specific genotype. In each MS run, the proficiency panel consisting of 43 samples with HPV plasmid dilutions in defined amounts (traceable to the International Standard for HPV DNA) was re-tested and negative controls (human DNA in Tris-EDTA buffer) included. The MS method was favourably assessed in the 2008 [22] and in the 2010 WHO Global HPV LabNet HPV DNA typing proficiency panel [23].

Among all types detected by MS-analysis, HPV68 was the type with the lowest probability to be detected (91%) when samples contained 200 copies per reaction. The detection probability was 26% when HPV68 was present in low copy numbers, i.e., 20 copies/reaction or in presence of other HPV types (detection probability = 17%) (JD, personal communication). Furthermore, the ME primer for HPV68 showed some cross-reactivity with HPV70 and consequently HPV68-positive samples were retested by using a Luminex-based detection with probes for HPV68A (GenBank accession number DQ080079), HPV68B (GenBank accession number M73258 for the original sequence ME180), and HPV70 for confirmation of the results [20].

### Statistical Analysis

The present analysis focused on 16 HPV types, including all 13 oncogenic types, i.e., HPV16, 18, 31, 33, 35, 39, 45, 51, 52, 56, 58, 59, and 68 [24] and two low-risk types, HPV6 and 11, targeted by one of the two currently available HPV vaccines. A logistic regression model was fitted with type-specific HPV positivity as an outcome, controlling for age (<20, 20–24, 25–29, 30–34, 35–39, 40–44, 45–49, ≥50 years), type of sample (urine only, genital swabs combined to urine, genital swabs only), and type-specific HPV prevalence, as previously described [2]. As the data have a hierarchical structure, with HPV infections nested within samples, multilevel models were used, with sample-level random effects. In this context, a random effect is an unobserved quantity that varies between samples and allows different levels of risk for prevalent HPV infection given the same observable risk factors. Random effects account for the fact that type-specific HPV measurements in the same sample are correlated with each other due to common risk factors acting at the sample level, notably sexual behaviour. A Bayesian approach with Markov Chain Monte Carlo simulation was used. Estimates were reported as posterior means and 95% credible intervals (95% CI). Discrepancies between the data (observed counts of co-infections per sample) and the model (expected counts of co-infections per sample) were assessed by posterior predictive two-sided p-values and measured by an observed-to-expected (O/E) ratio for each HPV co-infection [25]. All possible two-type interactions between the 16 HPV types were assessed and, therefore, 120 (16×15/2) statistical comparisons were generated for each sex. To minimize errors due to multiple comparisons, the Bonferroni correction was used to assess statistical significance. With 120 multiple comparisons for each sex, Bonferroni corrected p-value threshold for 2-type co-infections is 0.05/120 = 0.0004. For comparability with previous studies in which less conservative approaches had been used, we additionally reported associations that were significant by a p-value threshold of 0.01.

## Results

A total of 31,717 samples from women (mean age = 25.1 years, interquartile interval: 19–29 years) and 9,949 from men (25.3 years, interquartile interval: 20–29 years) were included in the present analysis. Type-specific prevalence for women and men was described in detail elsewhere [20]. Briefly, 38.5% of samples from women and 11.1% from men were HPV-positive in the HT-HPV study. In both sexes, HPV positivity was lower in first-void urine samples (27.2% in women; 10.5% in men) compared to combined samples with genital swabs immersed in first-void urine (44.9% in women; 22.2% in men) and to genital swab samples (43.0% in women; 21.3% in men) [20].

Table 1 shows the O/E ratios of women (1a) and men (1b) with single and multiple HPV infections. Multiple infections were detected in 41.9% of the 12,211 HPV-positive women and in 22.2% of the 1,104 HPV-positive men. The Basic model included only age, type of sample, and specific HPV prevalence as covariates. The Full model included, in addition to the covariates of the Basic model, sample-random effects. In the Basic model among women, there were fewer double infections (O/E ratio 0.95, 95% CI: 0.93–0.97), but more multiple infections with ≥3 HPV types than expected (O/E ratio 1.95, 95% CI: 1.88–2.03). In men, the O/E ratio for ≥2 HPV types was 2.58 (95% CI: 2.34–2.85). In the Full model, the O/E ratio among women was 1.12 (95% CI: 1.11–1.14) for 2-types and it decreased to 1.06 (95% CI: 1.03–1.09) for ≥3-type co-infections. Among men, the O/E ratio for ≥2-type co-infections accordingly to the Full model decreased to 1.09 (95% CI: 1.00–1.19).

**Table 1:** Observed-to-expected ratio of multiple human papillomavirus infections, according to two models, in women (a) and men (b), Sweden.

a)			Basic model		Full model	
No of HPV types	O	%	E^a^	O/E(95% CI)^a^	E^b^	O/E (95%CI)^b^
0	19,506	61.5	17,179.2	1.14(1.13–1.14)	19,196.8	1.02 (1.01–1.02)
1	7,091	22.4	10,161.8	0.70(0.69–0.70)	7,855.5	0.90 (0.89–0.91)
2	3,246	10.2	3,415.6	0.95(0.93–0.97)	2,892.9	1.12 (1.11–1.14)
≥3	1,874	5.9	960.5	1.95(1.88–2.03)	1,771.7	1.06 (1.03–1.09)
**b)**			**Basic model**		**Full model**	
**No of HPV types**	**O**	**%**	**E** a	**O/E (95% CI)** a	**E** b	**O/E (95%CI)** b
0	8,845	88.9	8,612.6	1.03(1.02–1.03)	8,837.4	1.00 (0.99–1.01)
1	859	8.6	1,241.2	0.69(0.66–0.73)	886.7	0.97 (0.92–1.03)
≥2	245	2.5	95.2	2.58(2.34–2.85)	224.9	1.09 (1.00–1.19)

HPV: human papillomavirus; O: observed; E: expected; CI: credible interval.

a Adjusted for age, type of sample, and type-specific HPV prevalence;

b As^a^ plus sample random effects.

In appendix (Table S1), we present analyses for women stratified according to the type of sample. With the Basic model, the O/E ratio for infections with ≥3 HPV types was greater in urine samples only (3.04, 95% CI: 2.80–3.29) than in urine plus genital samples (1.79, 95% CI: 1.72–1.86). With the Full model, however, the two O/E ratios were similar for the two types of samples (1.05, 95% CI: 0.99–1.12; and 1.06, 95% CI: 1.02–1.09, respectively).

**Figure 1:**
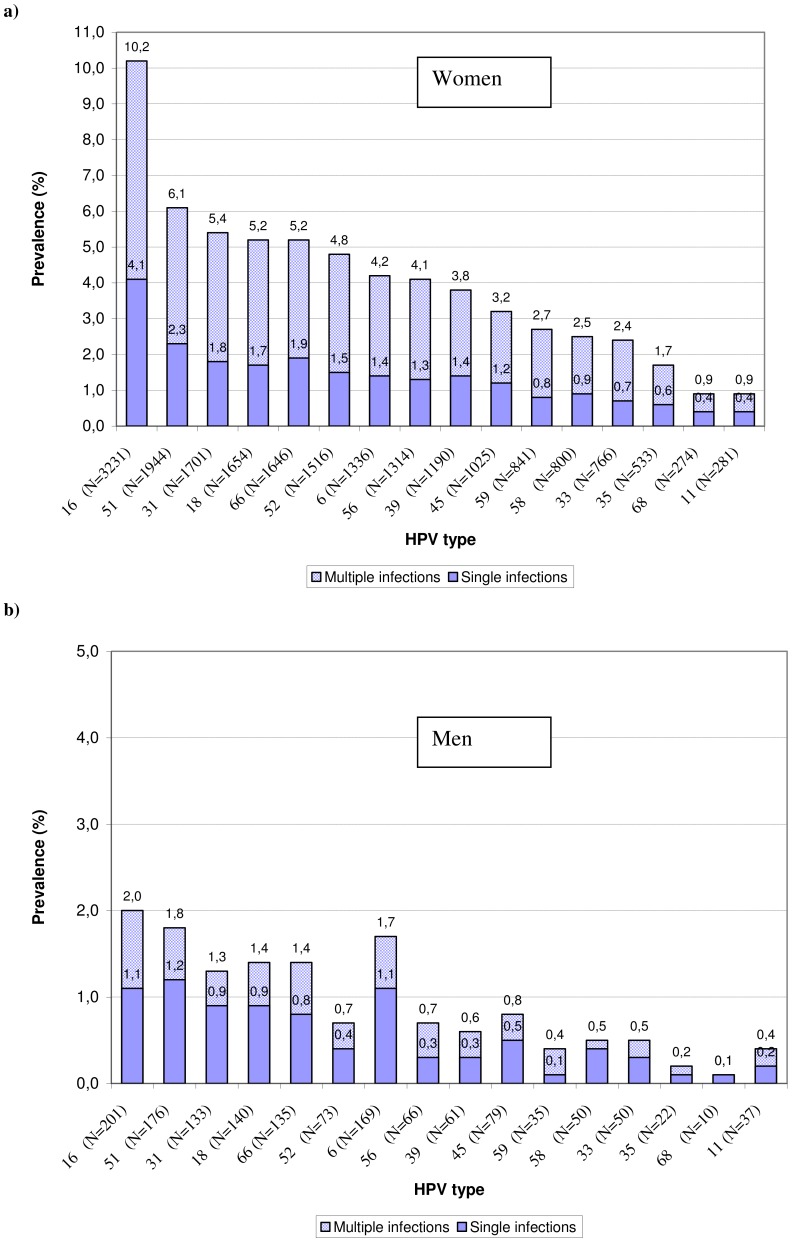
Type-specific human papillomavirus prevalence in samples from women (a) and men (b), Sweden.

In women, the proportion of HPV types involved in multiple infections ranged between 58.4% for HPV11 to 70.0% for HPV33 (Figure 1A). In men, the proportion of HPV types involved in multiple infections ranged between 30.0% for HPV58 to 62.9% for HPV59 (Figure 1B).

Table 2 shows the significant associations observed between 2-type combinations of HPV types in each sex, under the Full model. Only 3 associations reached the Bonferroni level of significance in women: HPV6/18 was found more often and HPV51/68 and 6/68 less often than expected. In men, none of the 2-type co-infections reached the level of significance of 0.0004. When a p-value threshold of p<0.01 was used, a few additional 2-way co-infections reached the level of statistical significance: HPV18/35, 35/66, and 6/58 (less often) and 56/66 (more often) in women. In men, HPV33/56 and 16/59 were found more often than expected, (Table 2).

**Table 2:** Human papillomavirus types showing significant excess or deficit in 2-type co-infections, Sweden.

		Female samples^c^ (N = 31,717)				Male samples^d^ (N = 9,949)			
Level of significance^a^	Co-infection with HPV types^b^	O	E^e^	O/E^e^	P-value	O	E^e^	O/E^e^	P-value
<0.0004	**6/18**	198	146.4	1.35	0.00028	16	11.4	1.42	0.30214
(Bonferroni)	**51/68**	15	36.7	0.41	0.00030	0	0.9	0	0.77396
	**6/68**	7	26.0	0.27	0.00010	0	0.9	0	0.82234
<0.01	**56/66**	190	143.5	1.332	0.00050	6	4.7	1.31	0.68458
	**18/35**	34	60.8	0.56	0.00054	1	1.7	0.63	0.93284
	**35/66**	34	60.5	0.56	0.00050	1	1.6	0.65	0.96466
	**6/58**	49	73.8	0.67	0.00414	3	4.4	0.70	0.74580
	**33/56**	74	69.7	1.06	0.65868	7	1.9	3.86	0.00810
	**16/59**	164	172.0	0.95	0.60632	13	3.6	3.72	0.00076

N: number; HPV: human papillomavirus; O: observed; E: expected.

a Two thresholds of significance are considered: one based on the Bonferroni method, i.e, 0.0004, and 0.01;

b Results are displayed if statistically significant in either women or men;

c Urine, urine+genital or vagina, cervix, cervix+urethra, urethra;

d Urine, urine+genital, urethra;

e As estimated by the Full model.

**Figure 2:**
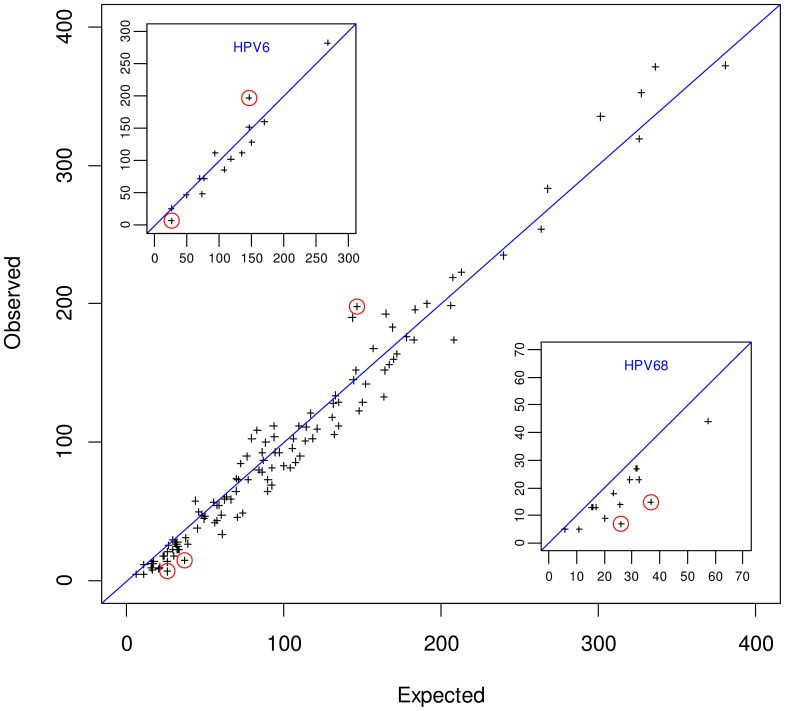
Observed *versus* expected occurrence for 2-type human papillomavirus infections, 31,717 samples from women, Sweden. Plus signs represent occurrences of HPV pairs. HPV pairs located in the upper triangle indicate positive clustering, while those located in the lower triangle represent negative clustering between the HPV types involved. Three of the p-values for joint HPV infections were significant at the chosen significance level of 0.0004: one positive clustering involving HPV6 with 18; two negative clustering involving the following pairs: HPV51/68, and HPV6/68. Overlaid on the main figure are the occurrences relative to HPV68 (small box on the bottom-right, scaled from 0 to 70) and to HPV6 (small box on the top-left, scaled from 0 to 300). To note, the significant negative cluster involving HPV6/68 appears on both small boxes. HPV68 showed a general tendency to be involved in negative clustering with all other types. The same behaviour was not observed for any other HPV type, including HPV6 that was also involved in 2 significant co-infections, but in opposite directions.

**Figure 3:**
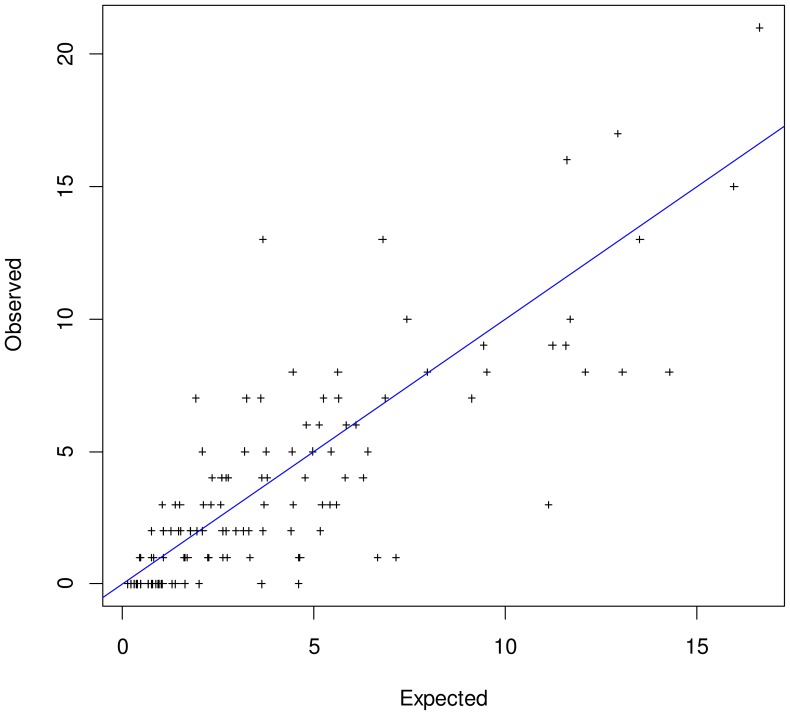
Observed *versus* expected occurrence for 2-type human papillomavirus infections, 9,949 samples from men, Sweden. Plus signs represent occurrences of HPV pairs. HPV pairs located in the upper triangle indicate positive clustering, while those located in the lower triangle represent negative clustering between the HPV types involved. There were no significant p-values for joint HPV infections at the chosen significance level of 0.0004.

Figure 2 shows the location for all 2-type co-infections of 16 HPV types across the diagonal drawn between the axis of the expected (horizontal) and observed (vertical) frequency in women. Plus signs represent HPV pairs. HPV pairs located in the upper triangle indicate positive clustering, while those located in the lower triangle represent negative clustering between the HPV types involved. As shown in Table 2, HPV6/18 was found significantly more often than expected while HPV6/68 and HPV51/68 were observed significantly less often than expected in 2-type co-infections. However, the majority of co-infections involving HPV68 also fell below the diagonal although the corresponding O/E ratios were not significantly different from unity (Figure 2, small bottom-right box). None of the other HPV types, including HPV6 (small top-left box), showed the same behaviour. Figure 3 reproduces the approach of Figure 2 for men and does not show any significant excess or deficit of 2-type infections.

## Discussion

Our large cross-sectional study of patterns of HPV types in multiple infections shows that, although HPV types often cluster together due to the common route of transmission, 2-type co-infections tend to occur at random in both sexes.

Our findings are thus consistent with previous studies from different areas of the world that showed, in both sexes, high frequency of multiple HPV infections but no significant excess or deficit of any 2-type combination [2], [3], [5]–[7], [9], [10], [16], [17]. In some instances, significant clusters were observed but they were attributable to technical problems. For instance, an analysis of the International Agency for Research on Cancer HPV Prevalence Surveys that used GP5+/6+ PCR assays to detect HPV, showed an excess of co-infections with HPV33/58, 18/45, 33/35, and 31/35 that was attributable to the cross-hybridization of highly homologous HPV types using enzyme immunoassay for genotyping [2]. Another analysis of the HPV in Men study found an apparent clustering of HPV52 with HPV35 or 58, which was due to the inability of the Roche Linear Array test to measure directly HPV52 positivity. HPV52 had, therefore, to be evaluated by subtraction using a mixed probe that also included HPV33, 35 and 58 [16].

The evaluation of HPV type patterns in multiple infections requires large studies on account of the need for multiple testing. Adequate statistical power is especially important when assessing the significance level of negative associations that involve rare HPV types. In the present study, there was no evidence of systematic clustering for most HPV types. Two of the three statistically significant associations in women involved HPV68, with either HPV6 or 51. However, HPV68 revealed a general tendency to be underrepresented in 2-type infections, although in most instances negative associations did not reach any of the two chosen levels of significance. The same behaviour was not observed for any other HPV type, including HPV6 that was also involved in two significant clusters: one more frequent and the other less frequent than expected. The most likely explanation of HPV68 pattern in multiple infections is a diagnostic artefact, on account of a less-than-optimal performance of the MS diagnostic method to detect HPV68, particularly where HPV68 is present in low copy numbers and in the presence of other HPV types.

Confirmatory testing of HPV68-positive samples with Luminex was performed to overcome cross-reactivity of HPV68 with 70 in the MS method. This step had the potential to improve the specificity but not the sensitivity of HPV68 testing in our study. Restriction to the original MS-based testing data did not make any difference (data not shown).

The excess of co-infections involving HPV6 and 18, two HPV types that are both targeted by current vaccines and that belong to different alpha species (alpha species 10 and 7, respectively), has no clear explanation, but we cannot exclude that it could be due to an extreme play of chance. An excess of co-infections, also emerged for genetically similar types HPV56 and 66, both belonging to alpha-6 species, but it was not statistically significant after Bonferroni correction.

Overall HPV prevalence in our study was higher in women (38.5%) compared to men (11.1%) [20]. The majority (>90%) of the samples from men in this study were urine only samples, whereas a combination sample with both genital swab and urine was most commonly used for women. Urine samples have a lower sensitivity to detect HPV than gold-standards (cervico-vaginal or penile swabs) and the problem is more severe in men (sensitivity estimates range between 28–56%) than in women (sensitivity estimates range between 84–91%) [26], [27]. The lower sensitivity in men can, at least partly, explain lower HPV prevalences compared to women and implies lower statistical power to detect significant HPV type clusters. Different sexual behaviours and, hence, different HPV prevalence in female and male participants to the *Chlamydia trachomatis* testing programme cannot be ruled out.

HPV testing in urine is affected by methods of collection, storage, DNA extraction, and the conservation medium used to avoid DNA degradation [28]. In our study, we used a validated protocol [20] and a commercial kit that included a buffer to stabilize DNA. Samples in which swabs were added to urine samples showed a similar HPV prevalence as those from genital swabs in both sexes [20], implying that sensitivity to detect HPV in genital swabs is not reduced after immersion in urine.

Genital swabs included in our study were collected in large majority from the cervix in women and the urethra in men and, therefore, our findings in swabs are likely to reflect the patterns of HPV co-infections in these genital sites. The comparison of findings in samples from women that included genital swabs with urine samples showed that, despite lower HPV-positivity in urine, the patterns of multiple infections with HPV types were consistent in the two sample types.

Information on sexual behaviour was not available in the HT-HPV study. Therefore, the inclusion of sample-level random effects was particularly useful in order to allow for unobservable risk factors common to all HPV types. Sample-level random effects, in fact, reduced substantially the O/E ratio for multiple HPV infections in both sexes. Sample-level random effects keep into account unobserved risk factors but do not allow to distinguish the effect of sexual behaviour from that of other risk factors, e.g., immunological susceptibility. The small excess of multiple HPV infections, present even after the inclusion of random effects, has also been observed in previous studies in both genders [2], [10], [11], [16]. We cannot exclude that it could be due to diagnostic artifacts or, alternatively, to some weak residual tendency of specific type combinations to cluster together that is not detectable unless we aggregate overall 2-type combinations.

A further limitation of the present study is that a proportion of samples, estimated to be around 22%, were repeat tests on the same individuals. As samples were anonymized, we could not identify repeat samples and allow for correlated measures, but we assumed that they were randomly distributed across subjects. Should that not be the case, we, however, expect that the use of random effects will prevent point estimates of O/E ratios from being substantially affected by the presence of repeated samples. Credible intervals, however, may be smaller than in the case of independent tests but this limitation should, if anything, lead to a larger number of significantly increased 2-type co-infections.

In conclusion, understanding whether certain HPV types have a tendency to cluster together in unvaccinated populations is important to evaluate the potential for type-replacement and cross-protection in HPV vaccine recipients and to understand better the natural history of the infection. Our study confirms in both sexes the lack of evidence of negative and positive clustering between types included in the current prophylactic HPV vaccines and other untargeted oncogenic types. In the present analysis, HPV6/68 and HPV51/68 were observed significantly less often than expected, but that was part of a general tendency of HPV68 to be underrepresented in combination with all other types. Lack of multiple infections involving HPV68 was likely to be due to a less-than-optimal performance of the MS primer for this type. The elimination of some HPV types by vaccination is thus unlikely to have any major effects on the occurrence of other HPV types.

## Supporting Information

Table S1 **Observed-to-expected ratio of multiple human papillomavirus infections, according to two models: in urine samples only (a); genital swabs only and genital swabs immersed in first-void urine (b), in women, Sweden.** HPV: human papillomavirus; O: observed; E: expected; CI: credible interval. ^a^Controlling for age and type-specific HPV prevalence; ^b^As a plus sample random effects.
(DOC)
