# Supplementary material for: Patterns of Human Papillomavirus Types in Multiple Infections: An Analysis in Women and Men of the High Throughput Human Papillomavirus Monitoring Study
Source: PLoS One. 2013 Aug 19;8(8):e71617. doi: 10.1371/journal.pone.0071617 (PMC3747214; doi:10.1371/journal.pone.0071617)
Supplement: Table S1 — Observed-to-expected ratio of multiple human papillomavirus infections, according to two models: in urine samples only (a); genital swabs only and genital swabs immersed in first-void urine (b), in women, Sweden. HPV: human papillomavirus; O: observed; E: expected; CI: credible interval. aControlling for age and type-specific HPV prevalence; bAs a plus sample random effects. (DOC) [file pone.0071617.s001.doc]

**Patterns of human papillomavirus types in multiple infections:**

**an analysis in women and men**

**of the High Throughput Human Papillomavirus Monitoring Study**

Salvatore Vaccarella^1*^, Anna Söderlund-Strand^2^, Silvia Franceschi^1^,

Martyn Plummer^1^, Joakim Dillner^1,2,3^

# Table S1. Observed-to-expected ratio of multiple human papillomavirus infections, according to two models: in urine samples only (a); genital swabs only and genital swabs immersed in first-void urine (b), in women, Sweden.

# a)

|  |  |  |  | Basic model | | Full model | |
| --- | --- | --- | --- | --- | --- | --- | --- |
| No of HPV types |  | O | % | E^a^ | O/E (95%CI)^a^ | E^b^ | O/E (95%CI)^b^ |
|  |  |  |  |  |  |  |  |
| 0 |  | 7,735 | 72.8 | 7,008.3 | 1.10 (1.09-1.12) | 7,652.4 | 1.01 (1.00-1.02) |
| 1 |  | 1,792 | 16.9 | 2,816.6 | 0.63 (0.63-0.65) | 1,996.1 | 0.90 (0.87-0.92) |
| 2 |  | 710 | 6.7 | 671.8 | 1.06 (1.01-1.11) | 607.4 | 1.17 (1.13-1.20) |
| 3+ |  | 387 | 3.6 | 127.3 | 3.04 (2.80-3.29) | 368.1 | 1.05 (0.99-1.12) |
|  |  |  |  |  |  |  |  |

# b)

|  |  |  |  | Basic model | | Full model | |
| --- | --- | --- | --- | --- | --- | --- | --- |
| No of HPV types |  | O | % | E^a^ | O/E (95%CI)^a^ | E^b^ | O/E (95%CI)^b^ |
|  |  |  |  |  |  |  |  |
| 0 |  | 11,771 | 55.8 | 10,186.4 | 1.16 (1.14-1.17) | 11,546.4 | 1.02 (1.01-1.03) |
| 1 |  | 5,299 | 25.1 | 7,313.7 | 0.72 (0.72-0.73) | 5,818.5 | 0.91 (0.90-0.92) |
| 2 |  | 2,536 | 12.0 | 2,760.8 | 0.92 (0.90-0.94) | 2,323.9 | 1.09 (1.07-1.11) |
| 3+ |  | 1,487 | 7.1 | 832.1 | 1.79 (1.72-1.86) | 1404.3 | 1.06 (1.02-1.09) |
|  |  |  |  |  |  |  |  |

HPV: human papillomavirus; O: observed; E: expected; CI: credible interval.

^a^Controlling for age and type-specific HPV prevalence;

^b^As ^a^ plus sample random effects.
